# Synergistic antiproliferative and pro-apoptotic effects of *Prunus armeniaca* and bee venom on breast cancer cells

**DOI:** 10.3389/fonc.2025.1647710

**Published:** 2025-08-25

**Authors:** Sultan F. Kadasah, Abdulmajeed F. Alrefaei, Fahd Mohammed Abd Al Galil, Abdulaziz M.S. Alqahtani

**Affiliations:** ^1^ Department of Biology, Faculty of Science, University of Bisha, Bisha, Saudi Arabia; ^2^ Department of Biology/Genetic and Molecular Biology Central Laboratory (GMCL), Jamoum University College, Umm Al Qura University, Makkah, Saudi Arabia

**Keywords:** breast cancer, *Prunus armeniaca*, bee venom, apoptosis, cell invasion

## Abstract

**Introduction:**

Breast cancer is the most prevalent malignancy among women worldwide, with triple-negative breast cancer (TNBC) posing significant therapeutic challenges due to its aggressive nature and lack of targeted treatments. Natural compounds such as *Prunus armeniaca* (PA) and bee venom (BV) have demonstrated anticancer potential.

**Methods:**

This study evaluates the synergistic effects of PA and BV on breast cancer cells, focusing on proliferation, apoptosis, and invasion. MCF-7 and MDA-MB-231 breast cancer cells were treated with varying concentrations (0–500 µg/mL) of PA, BV, and their combination. Cytotoxicity was assessed via the MTT assay, and the IC50 values were determined using GraphPad Prism. Colony formation, phase contrast microscopy, Acridine Orange/Ethidium Bromide (AO/EB) staining, transwell invasion, and Western blot assays were performed to evaluate proliferation, apoptosis, and invasion. Statistical significance was determined using one-way ANOVA.

**Result and discussion:**

The combination of PA and BV significantly enhanced cytotoxicity, with IC_50_ values reduced to 35.148 µg/mL in MCF-7 cells and 73.80 µg/mL in MDA-MB-231 cells, suggesting a synergistic effect. Colony formation assays revealed an 83% reduction at the highest dose (70.3 µg/mL). Morphological assessment showed characteristic apoptotic features, including cell shrinkage and membrane blebbing. AO/EB staining confirmed apoptosis induction, with apoptotic cells increasing from 3.2% in controls to 65.3% at 70.3 µg/mL. Western blot analysis demonstrated Bax upregulation and Bcl-2 downregulation, supporting apoptosis activation. Transwell invasion assays indicated a 59% reduction in cell invasion, suggesting that BV-PA effectively suppresses metastasis. BV-PA exhibits potent antiproliferative, pro- apoptotic, and anti-invasive effects in MCF-7 cells. These findings highlight its potential as a natural therapeutic strategy for breast cancer treatment, particularly TNBC. Further investigations are warranted to explore its molecular mechanisms and *in vivo.*

## Graphical Abstract

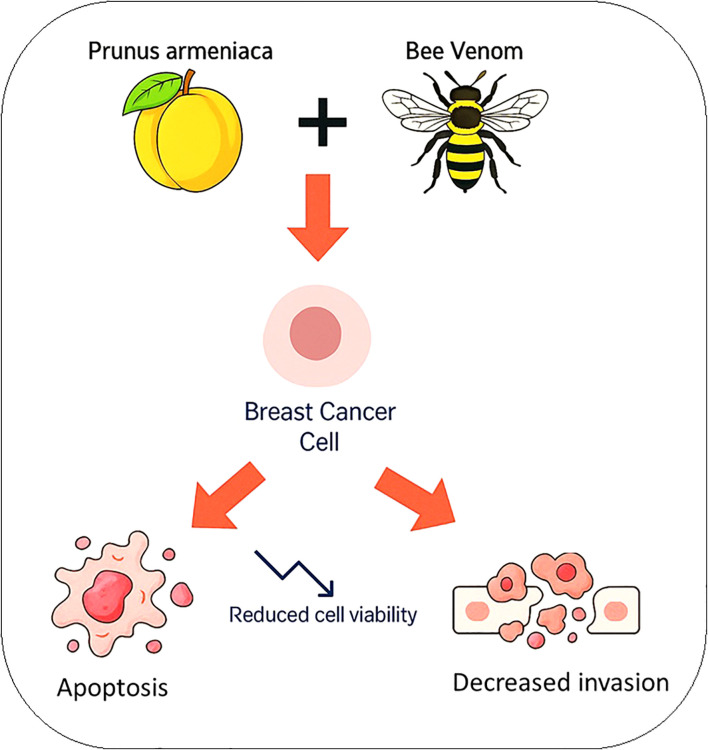

## Introduction

Cancer remains one of the most formidable health challenges and is the second leading cause of death worldwide, following cardiovascular disorders (1, 2). It is characterized by uncontrolled cell proliferation, invasion, and metastasis, all of which contribute to disease progression and poor prognosis. Early diagnosis remains essential for improving treatment success rates (3, 4). Although various therapeutic strategies—including chemotherapy, radiotherapy, gene therapy, surgery, and immunotherapy—are widely employed, the effectiveness of these approaches is frequently limited by systemic toxicity, drug resistance, and tumor heterogeneity (5, 6). Consequently, there is a pressing need to develop novel anticancer agents, particularly from natural sources, that offer improved efficacy with fewer adverse effects (7, 8).

Among all cancer types, breast cancer is the most frequently diagnosed malignancy in women and remains a major public health concern globally (9). According to GLOBOCAN 2022, there were an estimated 20 million new cancer cases and 9.7 million deaths worldwide. In the United States alone, 1,958,310 new cancer cases and 609,820 cancer-related deaths were projected for 2023 (10, 11). The high mortality rate of breast cancer is largely attributable to late-stage diagnosis, by which time metastasis has often occurred to critical sites such as the lymph nodes, brain, liver, lungs, or bones (12, 13). In 2022, approximately 2.3 million women were newly diagnosed with breast cancer, leading to 670,000 deaths worldwid**e** (14). Despite advances in screening and treatment, breast cancer remains a leading cause of cancer-related death among women. Breast cancer comprises various molecular subtypes, each differing in prognosis and treatment response. While triple-negative breast cancer (TNBC), which lacks ER, PR, and HER-2 expression, is known for its aggressive clinical course, the majority of experimental validation in this study was conducted on MCF-7 cells, which are estrogen receptor-positive (ER+) (11–13). Current therapeutic regimens for breast cancer include chemotherapeutic drugs such as doxorubicin, targeted agents like selective estrogen receptor modulators (e.g., raloxifene), monoclonal antibodies, and poly(ADP-ribose) polymerase (PARP) inhibitors such as olaparib (15, 16). However, these therapies often fail to provide lasting clinical benefit, necessitating the exploration of alternative or adjunct treatments (17).

Apoptosis, or programmed cell death, plays a vital role in maintaining tissue homeostasis by eliminating damaged or potentially malignant cells (18). Dysregulation of apoptotic signaling is a hallmark of cancer and commonly involves the overexpression of anti-apoptotic proteins such as Bcl-2 and the suppression of pro-apoptotic factors like Bax (19). The Bcl-2/Bax ratio is considered critical in determining a cell’s susceptibility to apoptosis, particularly in response to stress or chemotherapy (20). Natural products have gained attention for their potential role in cancer therapy due to their bioactivity, accessibility, and lower toxicity compared to conventional drugs (21). Apricot extract, derived from *Prunus armeniaca* (PA), has been reported to exert anticancer effects by downregulating anti-apoptotic genes and promoting apoptosis in various tumor cell lines (22–24). Similarly, bee venom (BV), traditionally used to treat ailments such as arthritis, asthma, and skin conditions, has shown promise in oncology research (25). BV contains a variety of bioactive components including melittin, apamin, and phospholipase A2, which have demonstrated selective cytotoxicity against cancer cells. Melittin, the principal peptide of BV, is known to disrupt cancer cell membranes and interfere with signaling pathways such as NF-κB and PI3K/AKT that are crucial for tumor cell survival and proliferation (26). In this study, we evaluated the cytotoxic effects of PA, BV, and their combination on breast cancer cells. Additionally, we assessed their synergistic effect on impact on apoptosis and cell invasion in TNBC cells.

## Materials and methods

### Cell culture

MCF-7 breast cancer cells (ATCC^®^ HTB22™) were obtained from VACSERA, Egypt. Cells were cultured in RPMI-1640 medium (Gibco, Invitrogen) supplemented with 10% fetal bovine serum (FBS) and antibiotics (100 U/mL penicillin, 100 mg/mL streptomycin). Chemicals, including PA extract (Prunus armeniaca) and bee venom (BV), were sourced from Sigma-Aldrich (USA) and VACSERA (Egypt), respectively.

### MTT assay

The antiproliferative effects of PA, BV, and their combination were assessed using the MTT assay. MCF- 7 and MDA-MB-231 cells were seeded in 96-well plates (4 × 10³ cells/well) and incubated for 24 hours before treatment with increasing concentrations (0–500 μg/mL) of PA, BV, and their mixture. After 72 hours, cell viability was evaluated using MTT reagent, and absorbance was measured at 570 nm using a microplate reader (BMGLABTECH FLUOstar Omega, Germany). IC50 values were calculated using GraphPad Prism.

### Colony formation assay

MCF-7 cells (500 cells/well) were seeded in six-well plates and treated with PA-BV (0, 70.3, 35.15, and 17.57 µg/mL) for 24 hours. The medium was replaced, and cells were incubated for 10–14 days. Colonies were fixed with 4% paraformaldehyde, stained with 0.5% crystal violet, and counted using ImageJ software.

### Phase contrast microscopy

Morphological alterations in MCF-7 cells following treatment with the PA-BV combination (0, 17.57, 35.15, and 70.3 µg/mL) were examined using an inverted phase contrast microscope (Olympus CKX53, Japan). Images were captured at 20× magnification to assess characteristic apoptotic features such as cell shrinkage, rounding, and detachment from the culture surface.

### Acridine Orange/Ethidium Bromide staining

Apoptotic changes were analyzed by AO/EB staining. MCF-7 cells were treated with PA-BV (0, 70.3, 35.15, and 17.57 µg/mL) for 24 hours, washed, and stained with AO/EB (1:1, 10 µg/mL). Cells were visualized under a fluorescence microscope (Olympus BX51, Japan) to distinguish live (green), early apoptotic (bright green), and late apoptotic (orange/red) cells.

### Transwell migration assay

Cell migration was evaluated using transwell inserts with an 8 μm pore size (Corning, USA). MCF-7 cells (5 × 10^4^ cells/well) were seeded in serum-free DMEM in the upper chamber, while DMEM supplemented with 10% FBS was placed in the lower chamber as a chemoattractant. Cells were treated with the PA-BV combination at concentrations of 0, 17.57, 35.15, and 70.3 µg/mL for 24 hours. After incubation, migrated cells on the lower surface of the membrane were fixed with methanol, stained with 0.5% crystal violet, and counted under an inverted microscope.

### Western blotting

MCF-7 cells were treated with PA-BV (0, 70.3, 35.15, and 17.57 µg/mL) for 24 hours, lysed using RIPA buffer with protease inhibitors, and centrifuged (13,000 rpm, 20 min, 4°C). Protein concentration was determined via the Bradford assay. Proteins (40 µg) were resolved on a 10% SDS-PAGE gel, transferred to a nitrocellulose membrane, and blocked with 5% skim milk in TBST. Membranes were incubated overnight at 4°C with primary antibodies against Bax, Bcl-2, and β-actin, followed by HRP-conjugated secondary antibodies. Protein bands were detected using an ECL detection system (Bio-Rad, USA), and densitometric analysis was performed using ImageJ software.

### Statistical analysis

All experiments were conducted in triplicate (n = 3) and results are presented as mean ± standard deviation (SD). Statistical analyses were performed using GraphPad Prism 8 (GraphPad Software Inc., USA). For comparisons between two groups, an unpaired two-tailed Student’s t-test was used. For experiments involving more than two groups, one-way analysis of variance (ANOVA) followed by Dunnett’s *post hoc* test was applied to determine statistical significance compared to the control group. A p-value of less than 0.05 was considered statistically significant. The number of independent biological replicates (n) for each assay is indicated in the corresponding figure legends.

## Results

### Synergistic antiproliferative effects of PA and BV in TNBC cells

**Figure 1:**
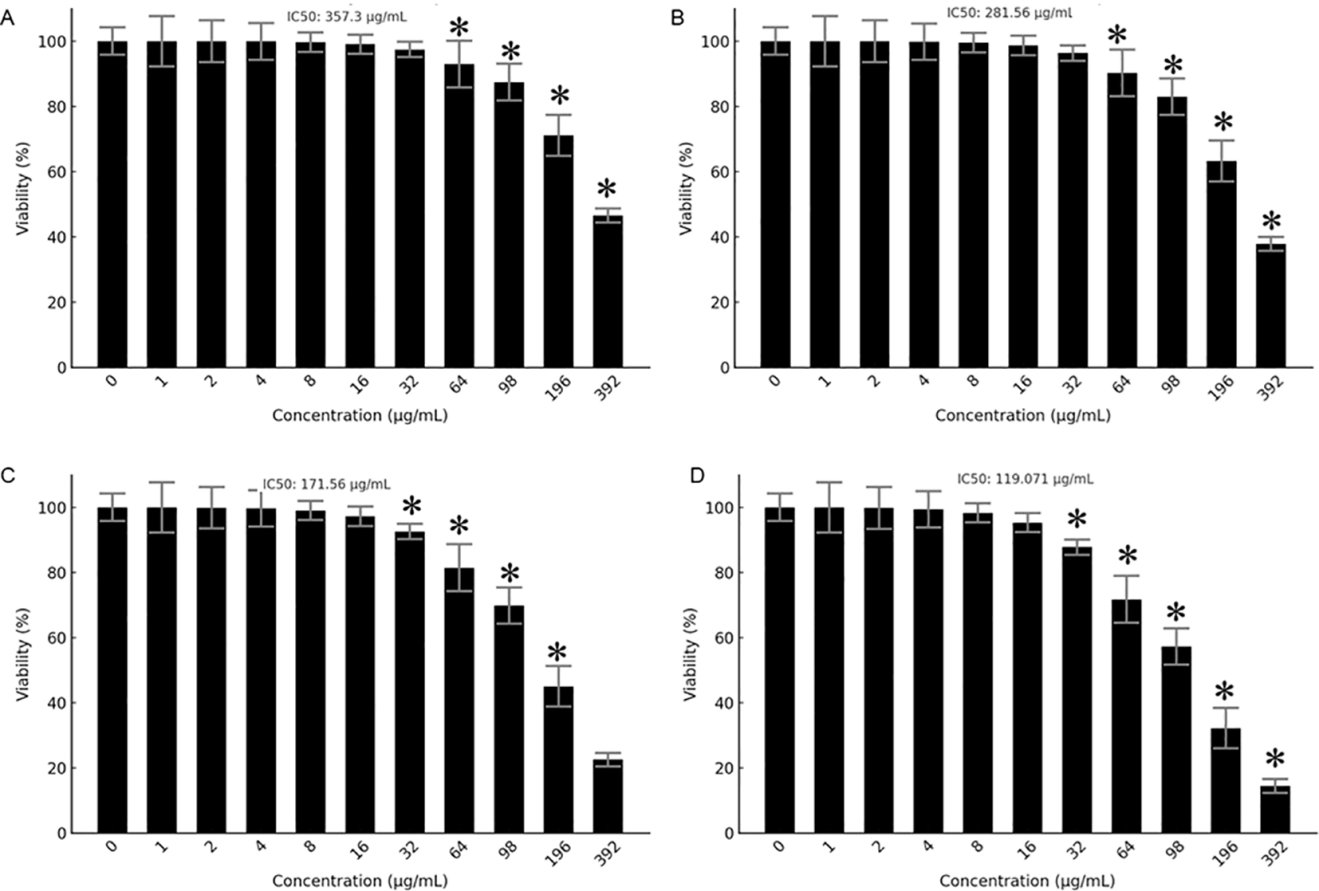
Antiproliferative effects of PA and BV on breast cancer cells. Effects of PA on **(A)** MDA-MB231 and **(B)** MCF-7 cells. Effects of BV on **(C)** MDA-MB231 and **(D)** MCF-7 cells. Experiments were conducted in triplicate and data is shown as mean ± SD (*P < 0.05).

**Figure 2:**
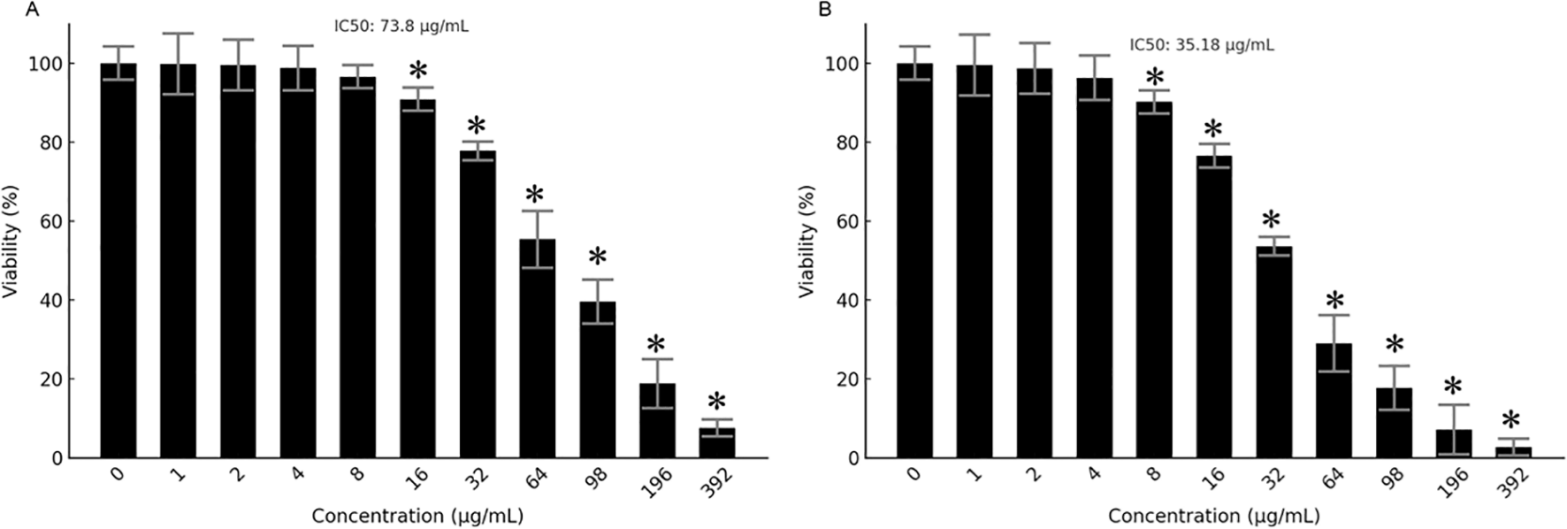
Antiproliferative effects of PA-BV mixture. MTT assay showing effects of PA-BV on **(A)** MDA-MB-231 cells and **(B)** MCF-7 cells. Experiments were conducted in triplicate and data is shown as mean ± SD (*P < 0.05).

The antiproliferative effects of Prunus armeniaca (PA), bee venom (BV), and their combination were evaluated in MCF-7 and MDA-MB-231 breast cancer cells using the MTT assay. The IC50 values for PA alone were 357.3 µg/mL in MDA-MB-231 and 281.56 µg/mL in MCF-7 cells, while BV alone exhibited IC50 values of 171.56 µg/mL in MDA-MB-231 cells and 119.071 µg/mL in MCF-7 cells (**Figures 1A–D**). Notably, the combination of PA and BV significantly enhanced cytotoxicity, reducing the IC50 values to 73.80 µg/mL in MDA-MB-231 cells 35.148 µg/mL in MCF-7 cells (**Figures 2A, B**). These findings suggest a synergistic interaction between PA and BV, leading to increased antiproliferative activity in TNBC cells, particularly in the highly invasive MDA-MB-231 cell line.

Since the combination of *Prunus armeniaca* (PA) and bee venom (BV) exhibited the lowest IC50 value of 35.148 µg/mL in MCF-7 cells, further experiments were conducted exclusively on this cell line to investigate its potential mechanistic effects on proliferation, apoptosis, migration, and protein expression. Moreover, concentrations of 17.57, 35.15, and 70.3 µg/mL were used in the subsequent assays correspond to approximately ½X, 1X, and 2X of the IC50 value in MCF-7 cells and were selected to represent a gradient of subtoxic to highly active doses for downstream assays.

### Effects of BV-PA on colony formation of MCF-7 cells

**Figure 3:**
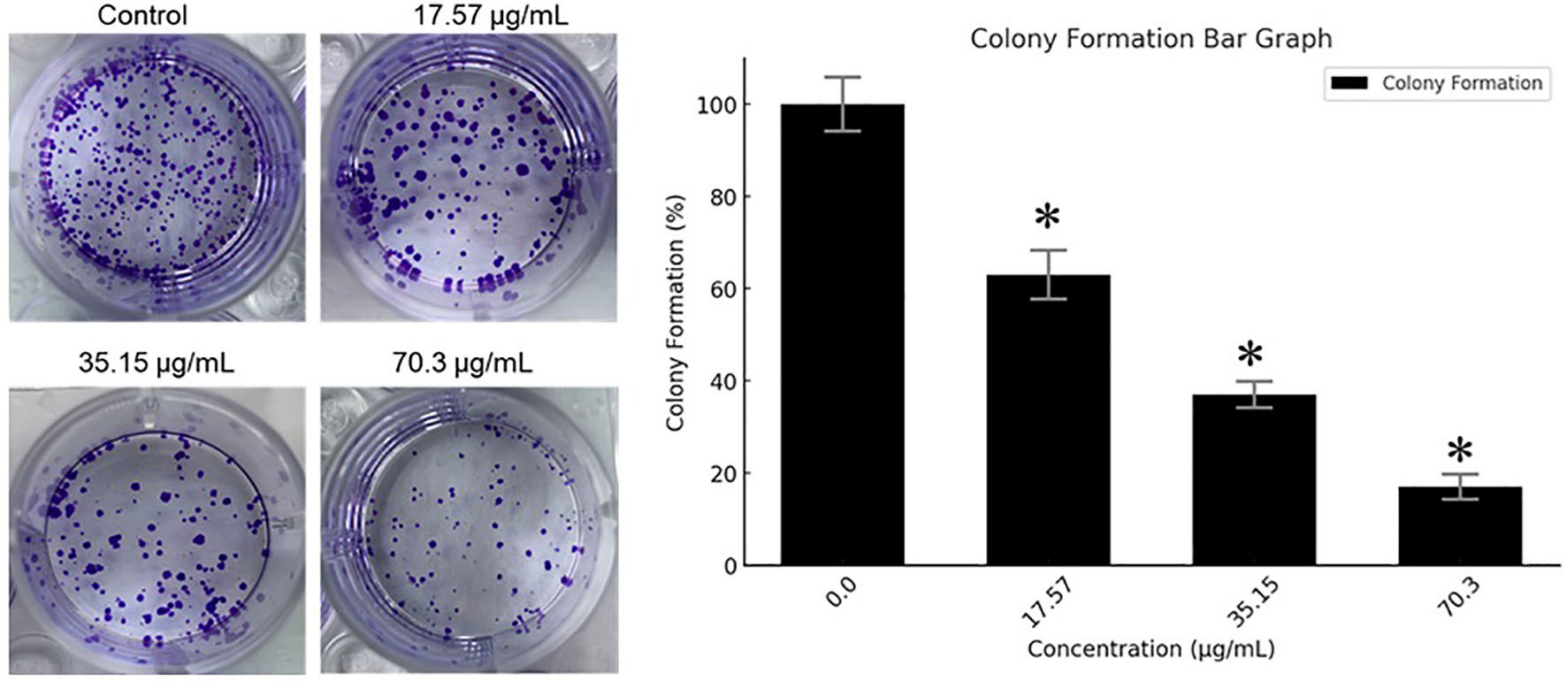
Effects of PA-BV on colony formation of MCF-7 cells. Experiments were conducted in triplicate and data is shown as mean ± SD (*P < 0.05).

The ability of MCF-7 cells to form colonies following treatment with the BV-PA mixture was assessed at concentrations of 17.57, 35.15, and 70.3 µg/mL. A concentration-dependent reduction in colony formation was observed, with the highest tested dose (70.3 µg/mL) resulting in an 83% inhibition compared to the control (**Figure 3**). These findings indicate that BV-PA effectively suppresses the long- term proliferative potential of MCF-7 cells.

### Effects of BV-PA on cell morphology of MCF-7 cells

**Figure 4:**
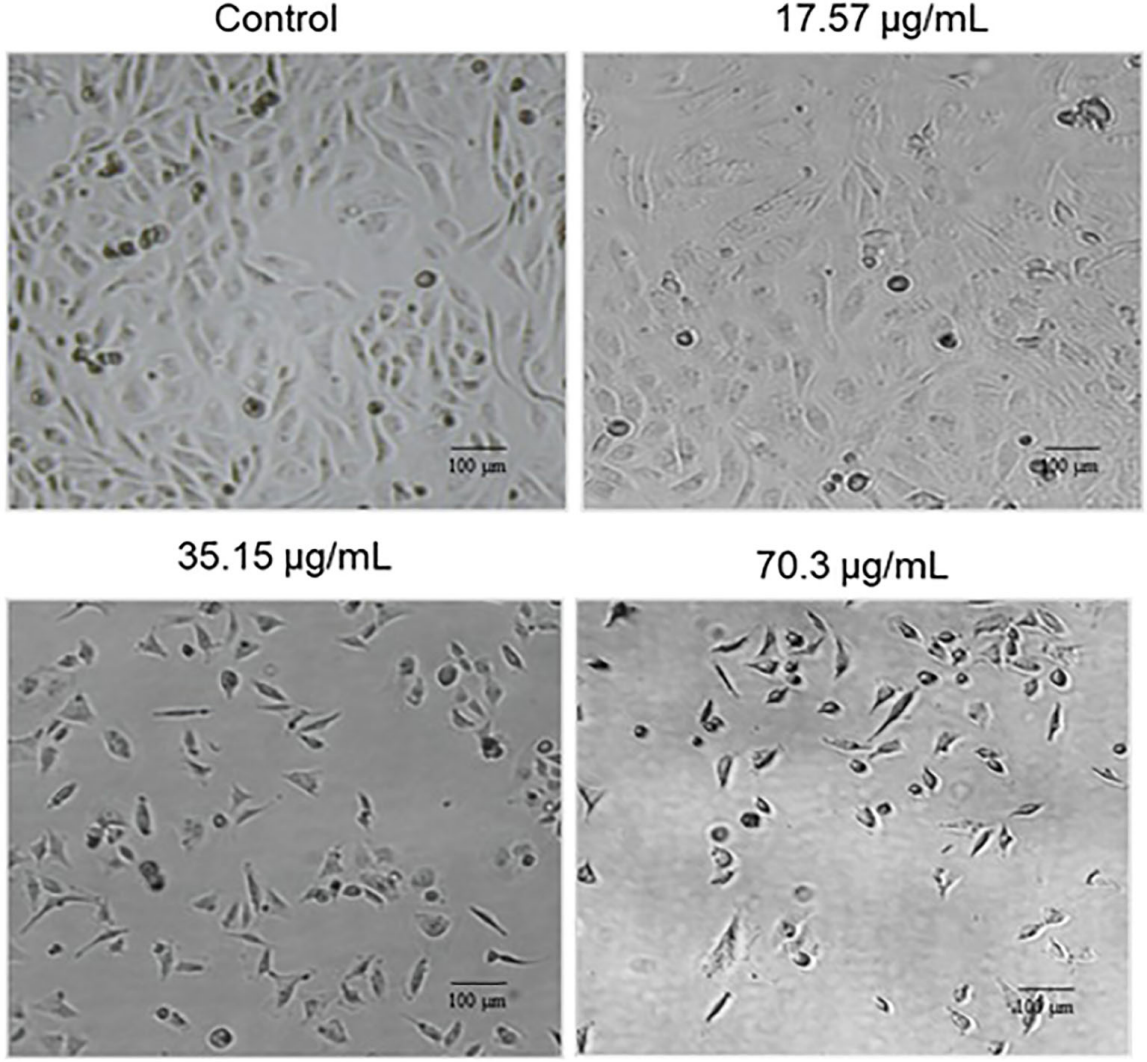
Phase contrast microscopy showing the effects of PA-BV on the cellular morphology of MCF-7 cells. Experiments were conducted in triplicate.

To evaluate the morphological alterations induced by BV-PA, MCF-7 cells were treated with 17.57, 35.15, and 70.3 µg/mL of the mixture and visualized using phase contrast microscopy. The treated cells exhibited signs of apoptosis, including cell shrinkage, membrane blebbing, and detachment from the culture surface (**Figure 4**). These morphological changes were more pronounced at higher concentrations, suggesting a dose-dependent apoptotic response induced by BV-PA in MCF-7 cells.

### BV-PA mixture induces apoptosis in MCF-7 cells

**Figure 5:**
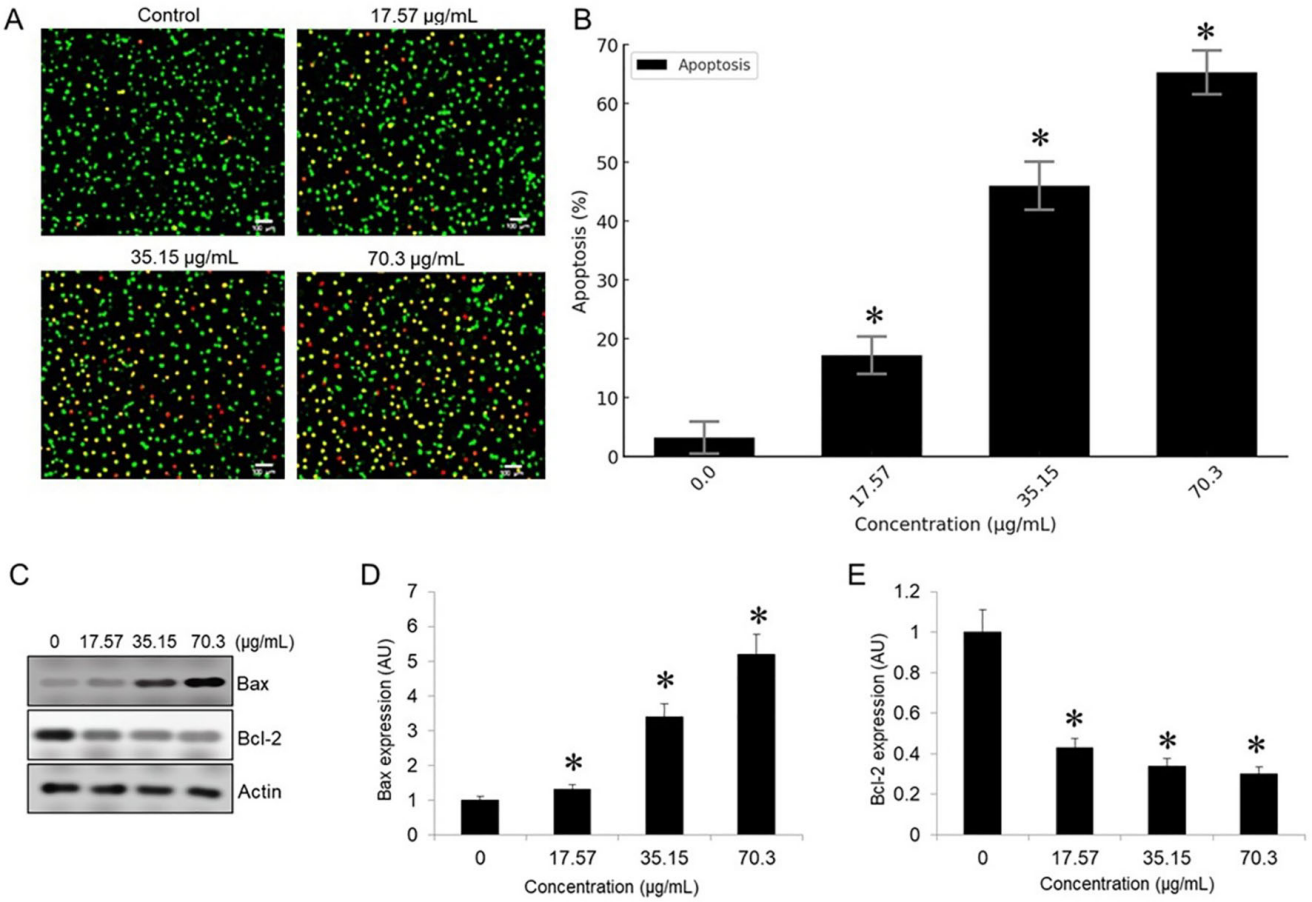
Figure X. PA-BV combination induces apoptosis in MCF-7 cells. **(A)** AO/EB staining shows increased apoptosis in MCF-7 cells treated with PA-BV (17.57–70.3 µg/mL) for 24 (h) **(B)** Bar graph showing quantification of apoptotic cells (%) **(C)** Western blot showing Bax, Bcl-2, and β-actin expression **(D)** Densitometric analysis of Bax and **(E)** Densitometric analysis of Bcl-2 normalized to β-actin shown in arbitrary units (AU). Data are mean ± SD (n = 3); *p < 0.05 vs. control.

To determine the apoptotic effects of BV-PA on MCF-7 cells, Acridine Orange/Ethidium Bromide (AO/EB) staining was performed. Fluorescence microscopy revealed distinct nuclear changes indicative of apoptosis. Live cells emitted green fluorescence due to intact membranes, while early apoptotic cells exhibited bright green fluorescence with condensed chromatin. Late apoptotic cells were distinguished by orange/red fluorescence, indicating membrane permeabilization and nuclear fragmentation. Treatment with BV-PA induced a significant increase in apoptotic cell populations, with apoptosis levels rising from 3.2% in the control to 65.3% at 70.3 µg/mL in a dose-dependent manner (**Figures 5A, B**). Additionally, Western blot analysis confirmed apoptosis by assessing Bax and Bcl-2 expression. Bax (pro-apoptotic protein) was upregulated, while Bcl-2 (anti-apoptotic protein) was downregulated, leading to a significant increase in the Bax/Bcl-2 ratio (**Figures 5C–E**). These results suggest that BV-PA promotes apoptosis in MCF-7 cells by activating pro-apoptotic signaling and inhibiting survival pathways.

### BV-PA inhibited invasion of MCF-7 cells

**Figure 6:**
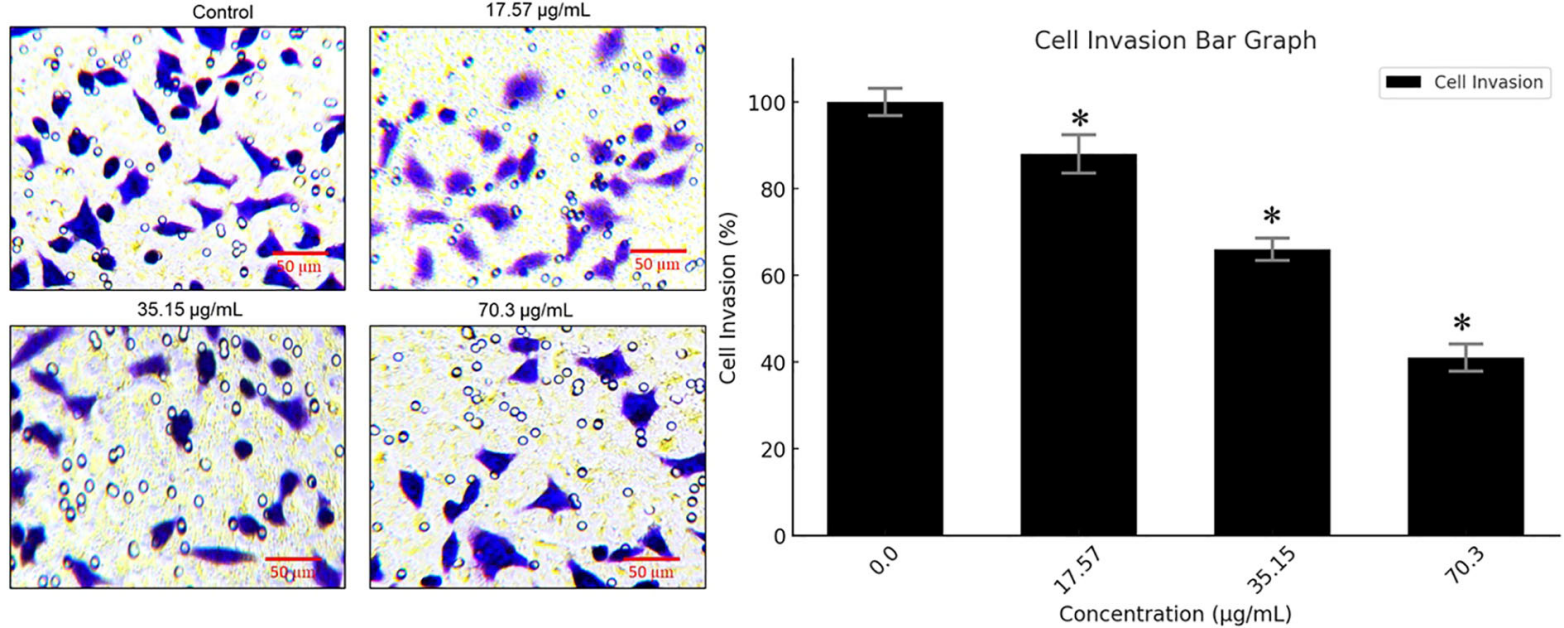
PA-BV inhibits invasion of breast cancer cells. Transwell assay showing effects of PA-BV on invasion of MCF-7 cells. Experiments were conducted in triplicate and data is shown as mean ± SD (*P < 0.05).

To assess the anti-metastatic potential of BV-PA, transwell invasion assays were performed on MCF-7 cells treated with 17.57, 35.15, and 70.3 µg/mL of the mixture. The results demonstrated a significant reduction in invasive capacity, with 59% inhibition at 70.3 µg/mL. This suggests that BV-PA effectively suppresses MCF-7 cell migration and invasion, highlighting its potential role in preventing breast cancer metastasis (**Figure 6**). These findings collectively underscore the potent antiproliferative, pro- apoptotic, and anti-invasive effects of the BV-PA mixture, demonstrating its promising therapeutic potential against breast cancer.

## Discussion

Breast cancer remains one of the leading causes of cancer-related mortality worldwide, with triple- negative breast cancer (TNBC) posing a particular challenge due to its aggressive nature and lack of targeted therapies (14). Current treatment options, including chemotherapy, radiotherapy, and immunotherapy, often exhibit limited efficacy due to tumor resistance and severe side effects (27). As a result, there is a growing interest in identifying alternative therapeutic approaches, particularly from natural compounds with potent anticancer properties. *Prunus armeniaca* (PA) and bee venom (BV) have been widely studied for their antitumor potential (22–27), with accumulating evidence suggesting that they can selectively target cancer cells while minimizing harm to normal tissues.

The present study demonstrated that PA and BV exerted a synergistic cytotoxic effect on MCF-7 cells, as evidenced by the significant reduction in IC_50_ values when used in combination. This enhanced cytotoxicity can be attributed to their complementary mechanisms of action. PA has been reported to induce apoptosis through mitochondrial-mediated pathways, while BV disrupts membrane integrity and modulates key apoptotic proteins such as p53, Bax, and Bcl-2 (28). The results of this study align with previous reports highlighting the ability of PA to suppress tumor growth by downregulating anti- apoptotic proteins and upregulating pro-apoptotic markers. Similarly, BV has been shown to activate apoptotic pathways via reactive oxygen species (ROS) generation and p53 activation, ultimately leading to cell cycle arrest and programmed cell death (25–27).

The inhibition of colony formation in MCF-7 cells further supports the long-term efficacy of PA-BV treatment in reducing the proliferative capacity of breast cancer cells. Notably, treatment with PA-BV led to an 83% decrease in colony formation at the highest concentration tested, suggesting that this combination therapy not only inhibits immediate cell growth but also prevents cancer cell survival and expansion over time. These findings are consistent with previous studies demonstrating that PA extract reduces colony-forming ability in various cancer cell lines (29).

Apoptosis, a key mechanism underlying the anticancer effects of PA and BV, was confirmed through AO/EB staining and Western blot analysis. The fluorescence-based AO/EB assay revealed a dose- dependent increase in apoptotic cells, with late-stage apoptosis reaching 65.3% at the highest concentration. Western blot results showed a marked upregulation of Bax and downregulation of Bcl- 2, leading to an increased Bax/Bcl-2 ratio, which is a well-established indicator of apoptosis induction (30). These results align with previous findings demonstrating that PA and BV modulate key apoptotic.

regulators in various cancer models. PA has been reported to activate the intrinsic apoptotic pathway by promoting cytochrome c release from mitochondria (28), while BV has been shown to enhance apoptosis by targeting TNF-α and disrupting cancer cell membrane integrity (31).

The observed synergistic effects of PA and BV may result from their complementary mechanisms of action at the molecular level. PA is rich in amygdalin and polyphenolic compounds, which can induce mitochondrial dysfunction, increase reactive oxygen species (ROS) generation, and promote cytochrome c release, leading to intrinsic apoptosis activation (22, 23, 28). BV, on the other hand, contains melittin and phospholipase A2, which disrupt cancer cell membranes, enhance cellular permeability, and initiate apoptotic signaling through activation of the p53 pathway and inhibition of PI3K/AKT/mTOR and NF-κB survival pathways (25, 26, 31–35). When used in combination, BV may enhance the intracellular uptake of PA by increasing membrane permeability, while concurrently inducing oxidative stress and mitochondrial destabilization. This dual assault on cancer cells—through both membrane disruption and mitochondrial targeting—may lead to amplified activation of apoptosis. Additionally, the simultaneous modulation of death receptor-mediated and mitochondrial pathways may overwhelm the cellular defense mechanisms and explain the pronounced synergistic effect observed.

Beyond apoptosis, this study also revealed that PA-BV treatment effectively inhibited the invasive potential of MCF-7 cells. Transwell migration assays demonstrated a 59% reduction in invasion following treatment, suggesting that PA and BV interfere with key molecular pathways involved in tumor metastasis. Previous research has indicated that BV inhibits epithelial-mesenchymal transition (EMT) by downregulating mesenchymal markers such as N-cadherin and vimentin, thereby reducing metastatic potential (36–38). Similarly, PA has been shown to suppress metastasis of cancer cells (39). The growing resistance to standard chemotherapy highlights the need for novel therapeutic strategies that can effectively target cancer cells while minimizing adverse effects (40). The findings of this study suggest that PA and BV may serve as promising candidates for complementary breast cancer therapy. Although, this study provides important preliminary evidence of the synergistic antiproliferative and pro-apoptotic effects of PA and BV on breast cancer cells. However, several limitations must be acknowledged. First, while both ER-positive (MCF-7) and triple-negative (MDA-MB-231) breast cancer cell lines were screened for cytotoxicity, subsequent mechanistic assays were conducted exclusively on MCF-7 cells. This choice was based on the lower IC50 observed in MCF-7 cells and their better morphological adherence for imaging-based assays. Nonetheless, the omission of mechanistic validation in MDA-MB-231 cells limits the generalizability of our findings to more aggressive TNBC models (41). Second, although the combination of PA and BV showed enhanced cytotoxicity compared to individual treatments, a formal synergy analysis (e.g., Chou–Talalay combination index) was not performed due to the experimental design constraints. Hence, the inference of synergism is based primarily on the reduction in IC50 values and should be interpreted cautiously. Third, all experiments were conducted *in vitro*, which does not fully capture the complexity of tumor–host interactions, systemic toxicity, or pharmacokinetics. The effects observed in MCF-7 cells may differ *in vivo* due to metabolic, immune, and stromal factors. Lastly, only a limited number of molecular markers (Bax and Bcl-2) were evaluated to assess apoptosis. Broader profiling of signaling would further enhance mechanistic understanding. Future studies are warranted to include additional cell lines, formal synergy modeling, and *in vivo* validation to confirm the therapeutic potential and safety of the PA-BV combination in breast cancer models.

## Conclusion

In conclusion, this study demonstrates that the combination of Prunus armeniaca (PA) and bee venom (BV) exerts potent antiproliferative, pro-apoptotic, and anti-invasive effects in MCF-7 breast cancer cells. These findings suggest that the PA-BV combination holds promise as a natural therapeutic strategy for breast cancer, including the challenging triple-negative subtype (TNBC), where treatment options remain limited. However, the mechanistic investigations were limited to MCF-7 cells, and further studies are needed to validate these effects in more aggressive TNBC models such as MDA-MB-231. Future research should focus on elucidating the molecular mechanisms involved—particularly the PI3K/AKT/mTOR and p53-mediated apoptotic pathways—and evaluating the efficacy and safety of this combination in *in vivo* breast cancer models to better understand its clinical relevance.

## Data Availability

The original contributions presented in the study are included in the article. Further inquiries can be directed to the corresponding author.
